# Identification of the complete mitochondrial genome of the king penguin *Aptenodytes patagonicus* (Sphenisciformes: Spheniscidae: Aptenodytes)

**DOI:** 10.1080/23802359.2019.1623121

**Published:** 2019-07-10

**Authors:** Jing Du, Jia-Shen Tian, Zhi-Chuang Lu, Sheng-Jiu Zhang, Xin-Ran Song, Gui-Ying Liu, Jia-Bo Han

**Affiliations:** a Dalian Key Laboratory of Conservation Biology for Endangered Marine mammals, Liaoning Ocean and Fisheries Science Research Institute, Dalian, Liaoning, China;; b Dalian Sun Asia Tourism Holding Co., Ltd, Dalian, Liaoning, China

**Keywords:** *Aptenodytes patagonicus*, complete mitochondrial genome, phylogenetic status

## Abstract

The complete mitochondrial genome of the king penguin *Aptenodytes patagonicus* was firstly determined. The mitogenome is 17,477 bp in length and contains 13 protein-coding genes (PCGs), 22 transfer RNA (tRNA) genes, 2 ribosomal RNA (rRNA) genes, and a control region. The total nucleotide composition is 31.0% A, 22.2% T, 33.1% C, and 13.8% G, with a total A + T content of 53.2%. The phylogenetic analysis demonstrates a close relationship between *A. patagonicus* and *A. forsteri*. These results provide fundamental information for further phylogeny and genetic studies on Aptenodytes genus.

King penguin (*Aptenodytes patagonicus*), the second largest species of the penguin order (Sphenisciformes), often occurs on several Antarctic and sub-Antarctic islands (Clucas et al. 2016). Despite the total population of *A. patagonicus* is estimated to be 2.23 million pairs worldwide and the number is increasing (Miller et al. 2010), the highly fragmented nature of the king penguin’s habitat precludes continuous population displacement due to the anthropogenic global environmental change (Le Bohec et al. 2008; Cristofari et al. 2018). Genetic approaches dramatically contribute to the management and conservation of wild species, such as species genome organization (Asakawa et al. 1995; Lei et al. 2010) and taxonomic clarifications (Sebastian et al. 2018). In the present study, we identified the complete mitochondrial genome of *A. patagonicus* and constructed the phylogenetic relationship among Spheniscidae species, aiming to provide fundamental information for its further phylogeny and genetic studies.

The blood sample was collected from an adult female *A. patagonicus* bred in Dalian Sun Asia Tourism Holding Co., Ltd. (sampling GPS location: 38°52′47″N, 121°34′8″E), and stored in a disodium EDTA tube (No. DL-190329) at −80 °C by the Conservation Biology Laboratory of Liaoning Ocean and Fisheries Science Research Institute, China. The complete mitochondrial genome was sequenced and annotated according to our previous study (Tian et al. 2019), with 16 pairs of primers (Sangon Biotechnology Co., Ltd., Shanghai, China) synthesized to amplify the whole mitogenome of *A. patagonicus*.

The complete mitochondrial genome of *A. patagonicus* is 17,477 bp (GenBank Accession Number: MK801135). It consists of 13 protein-coding genes (PCGs), 22 transfer RNA (tRNA) genes, 2 ribosomal RNA (rRNA) genes, and a control region. The overall base composition is 31.0, 33.1, 13.8, and 22.2% for A, C, G, and T, respectively. The heavy strand codes 28 genes and the light strand codes 9 genes. The 13 PCGs of *A. patagonicus* encode 3788 amino acids. Most PCGs start with a typical ATG codon, except for *COI* and *ND3* which use the initiation codon GTG and ATC, respectively. There are four kinds of termination codon of PCGs, namely TAA/TAG/AGG/incomplete T. In the *ND3*, there is an extra cytosine in the position 9713 of *A. patagonicus* mitogenome. The *12S rRNA* and *16S rRNA* are 978 bp and 1553 bp in length, respectively. Twenty-one tRNA genes have typical clover-leaf structures, except for *tRNA-Ser* (GCT). The control region is 1898 bp in length and located between *tRNA-Glu* and *tRNA-Phe* genes.

In order to ascertain the phylogenetic status of *A. patagonicus*, a phylogenetic tree involving *A. patagonicus* mitogenome and other 16 Spheniscidae species mitogenome available in GenBank database was constructed based on maximum likelihood (ML) analysis, rooted with two Gaviidae species (Figure 1). The phylogenetic tree shows that *A. patagonicus* is closely related to *A. forsteri* and it belongs to Aptenodytes genus, which is clustered together with other Spheniscidae species. The results are consistent with the conclusions drawn by fossils data in the previous study initiated by Chávez-Hoffmeister (2014).

## Figures and Tables

**Figure 1.**
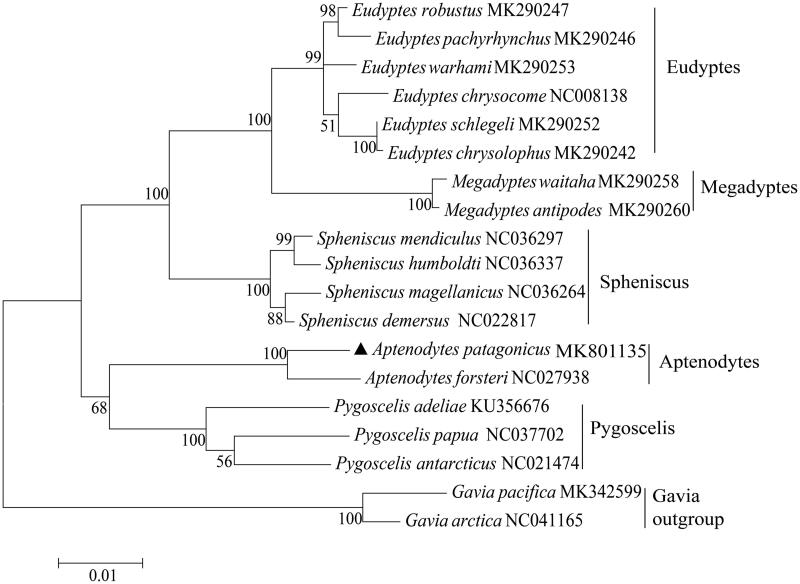
Inferred ML phylogenetic relationship of *A. patagonicus* between other Spheniscidae species based on the mitochondrial PCGs (amino acid data). Number above each node indicates the ML bootstrap support values generated from 100 replicates.
